# Loss of FADS2 Function Severely Impairs the Use of HeLa Cells as an *In Vitro* Model for Host Response Studies Involving Fatty Acid Effects

**DOI:** 10.1371/journal.pone.0115610

**Published:** 2014-12-30

**Authors:** Anke Jaudszus, Christian Degen, Stephan W. Barth, Martin Klempt, Wiebke Schlörmann, Alexander Roth, Carsten Rohrer, Helga Sauerwein, Konrad Sachse, Gerhard Jahreis

**Affiliations:** 1 Max Rubner-Institut (Federal Research Institute of Nutrition and Food), Department of Physiology and Biochemistry of Nutrition, Karlsruhe, Germany; 2 Institute of Nutrition, Department of Nutritional Physiology, Friedrich Schiller University of Jena, Jena, Germany; 3 Max Rubner-Institut, Federal Research Institute of Nutrition and Food, Department of Safety and Quality of Milk and Fish Products, Kiel, Germany; 4 Institute of Animal Science, Physiology & Hygiene Unit, University of Bonn, Bonn, Germany; 5 Friedrich-Loeffler-Institut (Federal Research Institute for Animal Health), Institute of Molecular Pathogenesis, Jena, Germany; Frankfurt University, Germany

## Abstract

**Scope:**

Established epithelial cell lines equipped with pattern recognition receptors such as the Toll-like receptor (TLR)-2 are common tools for immune response studies on invading pathogens, e.g. the obligate intracellular species of *Chlamydia*. Moreover, such models are widely used to elucidate fatty acid-mediated immune effects. In several transformed cell lines, however, unusual loss of metabolic functions was described. The cell lines A549 and HeLa are poorly characterized in this respect. Therefore, we comparatively assessed the metabolic capacity of A549 and HeLa prior to proposed application as *in* *vitro* model for fatty acid effects on chlamydial infection.

**Methodology/Principal Findings:**

We incubated both cell lines either with substrates (C18∶2*n*−6 or C18∶3*n*−3) or products (C18∶3*n*−6, C18∶4*n*−3) of fatty acid desaturase-2 (FADS2), and analysed the fatty acid profiles after 24 h and 72 h by gas chromatography. Based on these data, we suspected that the complete discontinuation of normal biosynthesis of long-chain polyunsaturated fatty acids (LC-PUFA) in HeLa was due to loss of FADS2 function. Consequently, prostaglandin E2 (PGE2) formation was less inducible by TLR2 stimulation in HeLa, likely as a result of not only insufficient supply of precursors but also weak cyclooxygenase-2 (COX-2) response. In accordance, *Chlamydia* infection rates were consistently lower in HeLa than in A549. Sequence analysis revealed no alteration within the *FADS2* gene in HeLa. The *FADS2* expression level, however, was significantly lower and, in contrast to A549, not regulated by C18∶2*n*−6. A549 exhibited regular fatty acid metabolism and enzyme functionality.

**Conclusions/Significance:**

Our data show that HeLa cells considerably differ from A549 at several stages of fatty acid metabolism. The poor metabolic potential of HeLa, mainly concerning FADS2 upstream of COX-2 function, calls into question whether these cells represent a good model to unveil fatty acid or downstream eicosanoid effects in the course of intracellular bacterial infection.

## Introduction

The cell lines A549, a human airway epithelial cell line, and HeLa, which originate from the human cervix epithelium, are widely used to represent the epithelium as the first barrier to encounter and defend microbial infection [1], [2]. Invading pathogens, such as the obligate intracellular species of *Chlamydia*, are recognized by prominent host defence receptors referred to as Toll-like receptors (TLR) [3]. TLR2, for instance, predominantly recognizes the chlamydial major outer membrane protein (MOMP), and is therefore considered to be critical for *Chlamydia*-mediated host cell activation and pathology [4]–[6]. Engagement of TLR2 immediately initiates downstream signaling towards COX-2-catalysed formation and release of *n*−6 LC-PUFA-derived eicosanoids to propagate a specific immune response [2], [7], [8]. An altered PUFA biosynthesis pathway entailing an insufficient supply of eicosanoid and downstream docosanoid precursors would therefore have serious consequences for adequate orchestration of the local immune response. Since *in* *vitro* models can only provide insights into a limited part of the *in* *vivo* situation, it is highly important to ensure appropriate functionality regarding the part that should be displayed. Immortalized cell lines with tendency for continuous growth have been recognized to be prone to loss of function [9], and little is known concerning the suitability of A549 and HeLa to serve as reliable *in* *vitro* models for such studies on immune responses involving fatty acids or their metabolites.

Most mammalian cells synthesize *n*−6 or *n*−3 LC-PUFA in a sophisticated anabolic cascade with alternating desaturation and elongation steps from dietary supplied C18∶2*n*−6 and C18∶3*n*−3 [10]. The initial and rate limiting step is catalysed by the *FADS2*-encoded Δ-6 desaturase that introduces a defined double bond into the acyl chain of its main substrates C18∶2*n*−6 and C18∶3*n*−3 to produce C18∶3*n*−6 and C18∶4*n*−3, respectively. Moreover, an alternative Δ-8 desaturase activity *via* C20∶2*n*−6 and C20∶3*n*−3 has also been linked to FADS2 [11]. Following chain elongation, the next desaturation reaction undertaken by FADS1 with Δ-5 activity is required to synthesize C20∶4*n*−6 and C20∶5*n*−3, which are further metabolized to docosaenoics, again with the help of FADS2. Finally, and catalysed by COX-2 activity, C20- and C22-PUFA give rise to highly bioactive derivatives such as eicosanoids and docosanoids, which play crucial roles, e.g., in inflammation and its resolution [12], [13].

Herein, we show that the loss of FADS2 function entailed a complete discontinuation of normal PUFA biosynthesis in HeLa. Consequently, PGE2 formation was less inducible in HeLa, likely as a result of not only insufficient supply of precursors but also weak COX-2 response. These results may serve as an explanation for the observation that *Chlamydia* infection rates were consistently lower and less stable in HeLa than in A549.

## Materials and Methods

### Cell culture

HeLa (cervix adenocarcinoma epithelial cell line; ATCC number: CCL-2) or A549 (type II alveolar adenocarcinoma epithelial cell line; ATCC number: CCL-185) were seeded in 25 cm^2^ tissue culture flasks at a density of 1.5×10^5^/mL. Cells were cultured for 24 h in a total volume of 6 mL DMEM/10% FBS alone or supplemented with either 33 µM C18∶2*n*−6, C18∶3*n*−3, C18∶3*n*−6, or C18∶4*n*−3 (all from Sigma-Aldrich, Taufkirchen, Germany). Free fatty acids were dissolved in sterile DMSO (Roth, Karlsruhe, Germany) to produce a 100 mM stock solution and stored under nitrogen in aliquots at −20°C. A corresponding volume of DMSO accounting for maximum 0.03% was added to the fatty acid free cultures. After 24 h (and 72 h for some experiments), cells were harvested and either processed for viability determination, fatty acid analysis, assessment of cell surface CD36, or gene expression and sequence analysis. For stimulation of COX-2-mediated PGE2 formation, 1.5×10^5^ cells/mL were seeded into 6-well plates and cultured for 20 h in the presence of either 33 µM C18∶2*n*−6 or C18∶3*n*−6. Subsequently, 0.1 µg/mL PAM2CSK4 (acts as TLR2 agonist [14], Cayla-Invivogen, Toulouse, France) or 1 ng/mL recombinant TNF-α (Life Technologies, Darmstadt, Germany) was added. Corresponding wells were 1 h preincubated with 2 µM of a selective COX-2 inhibitor (sc-58125) or 200 nM of a selective COX-1 inhibitor (sc-560; both Biomol, Hamburg, Germany) before stimulation. Control cultures contained DMSO vehicle. After 8 h, cells were detached, centrifuged and processed for flow cytometry or protein quantification. Supernatants were collected and stored at −20°C until PGE2 analysis. Before they were used in the experiments, cells were subcultivated at least six times after thawing. Cultures were held under standard conditions at 37°C, 5% CO_2_, 95% humidity. Finally, in both A549 and HeLa a DNA profiling using 8 different and highly polymorphic short tandem repeat (STR) loci was carried out in order to confirm authenticity of each cell line (Authentication service, Leibniz-Institute DSMZ, Braunschweig, Germany).

### Viability and absolute cell count determination

Viability was flow cytometrically measured by annexin-V/propidium iodide exclusion staining as described previously [15]. Absolute cell count was quantified by flow cytometry using counting beads (eBioscience, Frankfurt/Main, Germany).

### Fatty acid analysis

Total lipids were extracted from PBS-washed cell pellets using a methanol/chloroform mixture according to Bligh and Dyer [16]. Subsequently, a transesterification was performed by incubating samples with 0.5 N methanolic sodium hydroxide at 100°C for 10 min followed by methanolic boron trifluoride (10% w/w; Supelco, Bellefonte, PA, USA) treatment at 100°C for 5 min. Subsequently, fatty acid methyl esters (FAME) were extracted with *n*-hexan, separated in a gas chromatograph (GC 17a V3, Shimadzu, Kyoto, Japan) using a fused-silica capillary column with medium polarity (DB 225 MS, 60 m×0.25 mm i.d., 0.25 µm film thickness; Agilent Technologies, Santa Clara, USA) and detected downstream by a flame ionization detector (FID). GC conditions were as previously described [17]. Peak area integration was accomplished using GC solution software version 2.3 in comparison to previously measured reference standards (BR2, BR4, and ME93 from Larodan/CPS-Chemie, Aachen, Germany; Menhaden from Sigma-Aldrich, Taufkirchen, Germany; 463 and 674 from Nu-Chek-Prep, Elysian, USA).

### *FADS2* and *FADS1* expression analysis

First strand cDNA was synthesized from 2 µg extracted total RNA (Total RNA and protein isolation kit, Macherey-Nagel, Düren, Germany) using random oligo(dT) primers and reagents according to the conditions supplied in the Transcriptor First Strand cDNA Synthesis Kit from Roche Diagnostics (Mannheim, Germany). Samples were stored at −20°C until further use. Target primer sequences and probes were retrieved from the Universal Probe Library (UPL, Roche) and are listed in Table 1. Primers were purchased from Biomers (Ulm, Germany). PCR efficiencies for each primer pair were determined by serial cDNA dilutions. The 20 µl reaction mixture contained 5 µL cDNA, 3 µL water, 0.5 µL (0.5 µM final concentration) of each primer, 1 µL (0.1 µM) probe and 10 µL 1×Light Cycler480 Probe Master Mix (Roche). All reactions were performed in technical triplicates in the Light Cycler480 Instrument (Roche) with a PCR profile including an initial denaturation step of 10 min at 95°C followed by 45 amplification cycles each at 95°C for 10 s, 60°C for 30 s and 72°C for 1 s, and a terminal cooling period of 10 s at 40°C. Analysis of the PCR results was carried out with the Light Cycler480 Software (Roche). Expression levels were determined by calculating relative quantifications using the ΔΔCT-method with *TUBA1A* (α1-tubulin) as the reference gene.

**Table 1:** Primers and UPL probes used for real-time gene expression analysis.

Gene name	GenBank accession#	Nucleotide sequence (5′→3′)	UPL probes	Amplicon	PCR efficiency
*FADS2* *	NM_004265.3	FW: ttaacttccagattgagcacca	#60	nt 1286–nt 1351	1.87
		RV: gggcgatcttgtgtaagttgt			
*FADS1*	NM_013402.4	FW: cattttgtgattggccacct	#11	nt 891–nt 986	2.05
		RV: gtctttgcggaagcagttg			
*TUBA1A*	NM_006009.3	FW: cttcgtctccgccatcag	#58	nt 304–nt 398	1.94
		RV: ttgccaatctggacacca			

*Products on intended targets: transcript variants 1–3.

### Sequence analysis of *FADS2* in HeLa

For full-length sequencing encompassing the entire *FADS2* coding region in HeLa cells, DNA was extracted using the Cristal DNA extraction Kit (Bio Lab Products, Hamburg, Germany) according to the instructions. PCR was performed with *Pfu* polymerases (Fermentas, St. Leon Roth, Germany) with 0.3 µM of each primer and conditions as recommended by the manufacturer. After initial denaturation at 94°C for 2 min, 35 cycles each of 94°C for 30 s, 60°C for 20 s, and 72°C for 90 s were performed. Primers (TIB Molbiol, Berlin, Germany) used for the amplification are listed in Table 2. Subsequently, the PCR products were cloned into pJet1.2 (Fermentas) and sequenced using the sequencing primers pJet1.2-F or pJet1.2-R at LCG sequencing service (Berlin, Germany).

**Table 2:** Primers used for *FADS2* encoding sequence analysis.

Coding exon no.	Primer sequence (5′→3′)	Position in AP002380
1, tr. var. 2 and 3	FW: aatgggctcctttcctgctc	137,054-73
	RV: ggctcagggacctacccc	137,713-30
1, tr. var. 1	FW: ccgctggccttcgaaagat	148,918-36
	RV: agatgcactttccttccctacag	149,713-35
2	FW: gatcacaggagaccccgttt	158,653-72
	RV: cagttactggtcatggggcaag	158,966-87
3/4	FW: gcattggctgttgaaatggc	161,249-68
	RV: accctgtagcctcagagcac	161,807-26
5	FW: cctgaagtgctatccccgag	169,042-61
	RV: tgtcagctctctaggcttcct	169,477-97
6/7	FW: ggtgtgccctgagcagatag	177,906-25
	RV: ccagctgtgtccccaactc	178,537-55
8/9/10	FW: agagggcaggttgcacctaa	183,938-57
	RV: gcccccacattctgcattct	184,860-79
11/12	FW: atagagcacaaacgccctcc	185,935-54
	RV: catacaccagcctctcggac	186,777-96

tr. var. - transcript variants.

### Flow cytometry for determination of cyclooxygenases and CD36 expression

For intracellular quantification of cyclooxygenase protein expression, cells were permeabilized by washing with PBS/0.1% BSA/0.1% saponine and stained with Multicolor anti-human COX-1-FITC/anti-human COX-2-PE monoclonal antibodies (mAb; clones AS70/AS57, BD, Heidelberg, Germany) as described previously [18]. To assess surface expression of CD36, cells were stained with anti-human CD36-APC mAb (clone TR9, Abcam, Cambridge, UK). Non-specific fluorescence was controlled by incubation with isotype-matched antibodies. Samples were measured on a FACSCalibur flow cytometer and analysed using CELLQUEST software (BD).

### PGE2 formation

PGE2 formation was quantified in supernatants using a competitive enzyme immunoassay (Cayman, Ann Arbor, USA) with indicated sensitivity of 50 pg/mL and limit of detection at 15 pg/mL. Data are related to the protein content of the corresponding cells which was determined by employing the Lowry method using bovine gamma-globulin as standard [19]. The colorimetric assay was purchased from Bio-Rad (München, Germany).

### Assessment of *Chlamydia* infection rates

HeLa and A549 were seeded in 12-well plates at a density of 1.5×10^5^ cells/mL and cultured for 24 h. Monolayers were infected with *Chlamydia psittaci* strain DC15 (DSM27008) at a multiplicity of infection (MOI) of 4 as described [20]. The strain was obtained from the collection of the National Reference Laboratory for Chlamydiosis at Friedrich-Loeffler-Institut Jena, Germany (Head: Dr. K. Sachse). 48 h post infection (p.i.), cells were trypsinized, fixed with methanol and processed for flow cytometric quantification of chlamydial infection as previously described [21]. For immunofluorescence staining, cells were inoculated with strain DC15 at indicated MOI of 4 in shell vials on coverslips. 48 h p.i., methanol-fixed coverslips with monolayers were mounted on slides, stained with fluorochrome-labeled antibody against *Chlamydiaceae*-specific lipopolysaccharide (IMAGEN Chlamydia kit, Oxoid Limited, Wesel, Germany) and visualized using fluorescence microscopy, according to Goellner *et al.* [20].

### Statistics

Based on likelihood ratio test (LRT), a linear model with generalized least squares was used to determine the relationship between the outcomes FAME and *FADS2* mRNA expression, respectively, and the two factors cell line (HeLa and A549) and fatty acid (FA) treatment (without and with) or the interaction of these factors. This model was considered appropriate because of variance inhomogeneity within the factor FA treatment. For comparison of data on COX-2 expression and PGE2 formation, the stimulation condition was entered as third independent factor. To avoid heteroscedasticity, data were log-transformed. Post hoc test was conducted using Tukey-Kramer test and p values were adjusted for multiple comparisons. All calculations were carried out using the gls-function from R package nlme version 3.1–103. Data on FADS ratios and infection rates were evaluated using 2-tailed Student’s t-test (SPSS software version 19.0, SPSS Inc., Illinois USA). To indicate differences of desaturation activity between HeLa and A549, the following product to substrate ratios were estimated and compared: (C18∶3*n*−6+C20∶3*n*−6)/C18∶2*n*−6 and (C18∶4*n*−3+C20∶4*n*−3)/C18∶3*n*−3 for FADS2 function (after 24 h incubation with C18∶2*n*−6 and C18∶3*n*−3, respectively), and C20∶4*n*−6/C20∶3*n*−6 and C20∶5*n*−3/C20∶4*n*−3 for FADS1 activity (after 24 h incubation with C18∶3*n*−6 and C18∶4*n*−3, respectively). Unless otherwise indicated, data are expressed as means ± SEM of four independent experiments that were performed based on different cryopreserve lots. Significance of difference was set at p≤.05.

## Results and Discussion

### Effect of fatty acid incubation on viability and cell growth

In both HeLa and A549 cell lines, there was no negative effect of any fatty acid treatment on viability and cell growth over an incubation period of 24 h to 72 h compared to the DMSO-control (data not shown).

### PUFA biosynthesis

**Figure 1:**
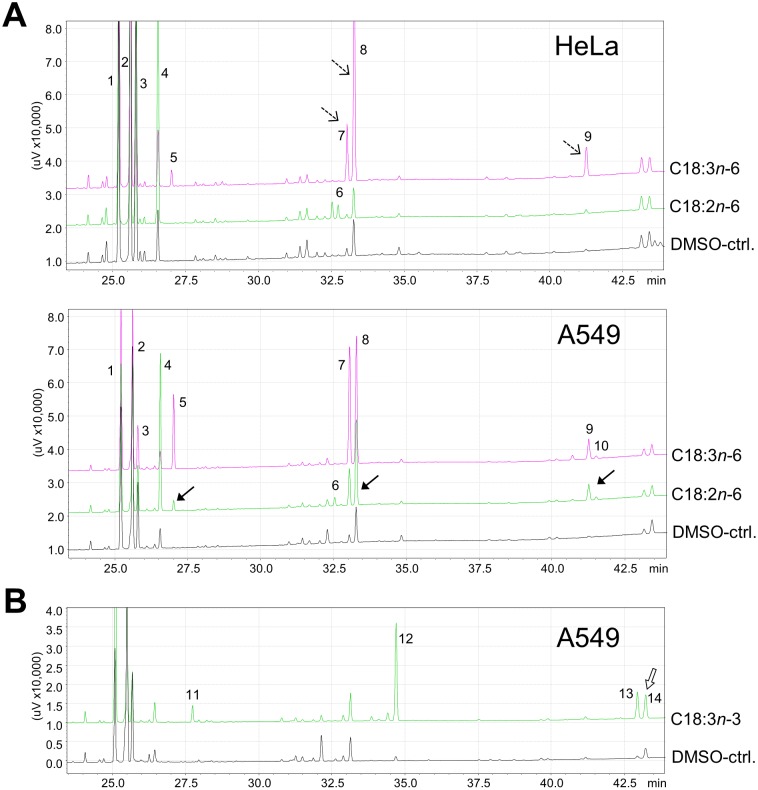
Partial chromatograms of cellular lipids of HeLa and A549. Cells were incubated without (DMSO-treated control) or with the indicated fatty acid (33 µM) for (**A**) 24 h or (**B**) for 72 h. 1–C18∶0, 2–C18∶1*c*9, 3–C18∶1*c*11, 4–C18∶2*n*−6, 5–C18∶3*n*−6, 6–C20∶2*n*−6, 7–C20∶3*n*−6, 8–C20∶4*n*−6, 9–C22∶4*n*−6, 10–C22∶5*n*−6, 11–C18∶3*n*−3, 12–C20∶5*n*−3, 13–C22∶5*n*−3, 14–C22∶6*n*−3. **A:** C20∶2*n*−6 (peak 6) was the only metabolite identified in HeLa following incubation with C18∶2*n*−6. In contrast, all detectable elongated and desaturated products of C18∶2*n*−6 increased in A549 (filled-headed arrows). When C18∶3*n*−6 was the supplement, the respective metabolites increased in HeLa, too (broken-lined arrows). **B:** As a product of C18∶3*n*−3, C22∶6*n*−3 (peak 14) increased in A549 after 72 h.

**Figure 2:**
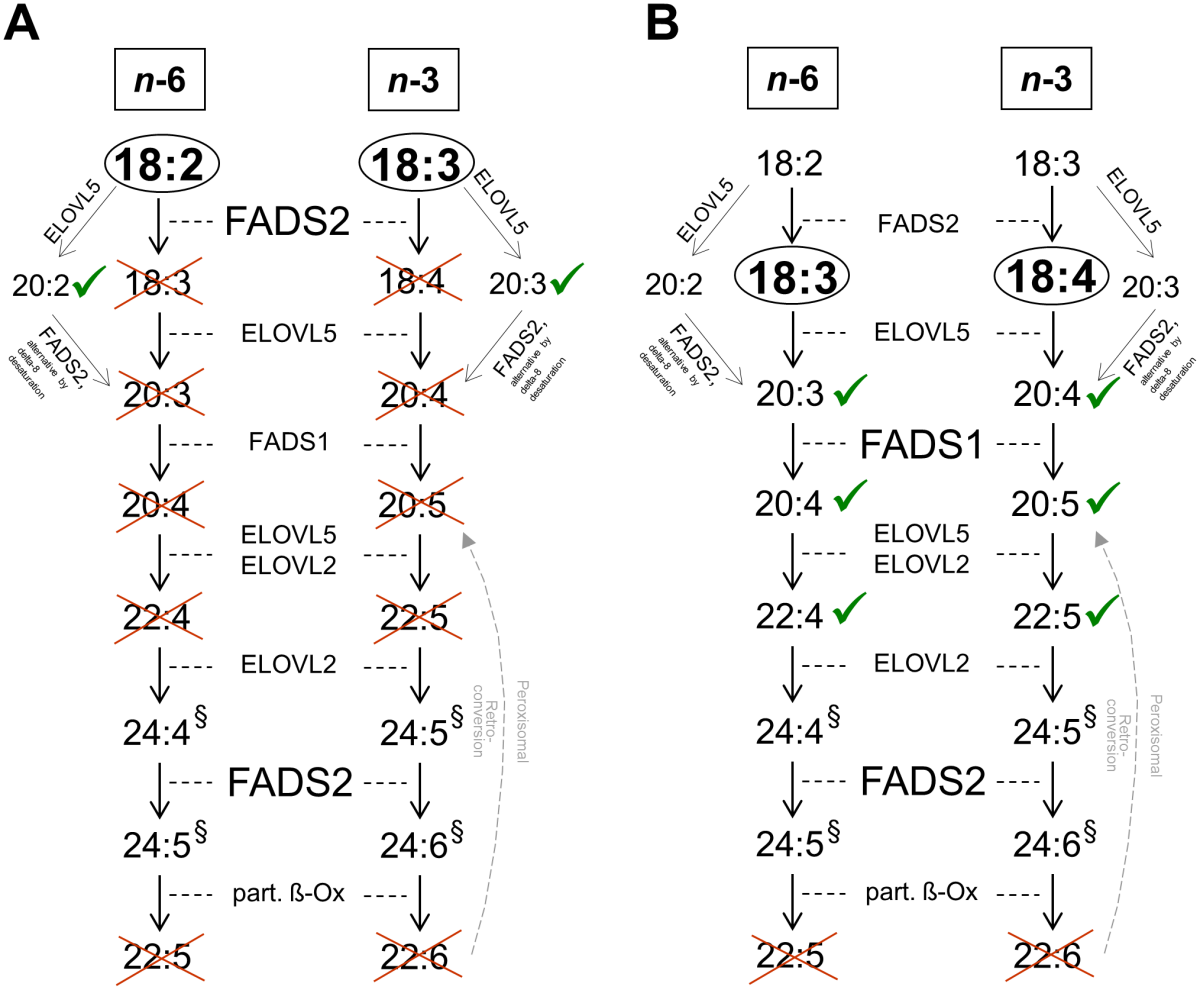
HeLa lack FADS2 but not FADS1 function. **A:** HeLa failed to metabolize C18∶2*n*−6 and C18∶3*n*−3 to their respective Δ-6 desaturation products. However, the respective elongation products increased. **B:** Bypassing the (first) FADS2-dependent step by incubation with C18∶3*n*−6 and C18∶4*n*−3, respectively, led to synthesis of all subsequent products, including those involving FADS1, up the second step that required FADS2 function. ^§^C24-LC-PUFA were not detectable due to methodological limitations. Ticked: product emerged, crossed: product not generated. ELOVL – fatty acid elongase, part. ß-Ox – partial ß-oxidation.

**Figure 3:**
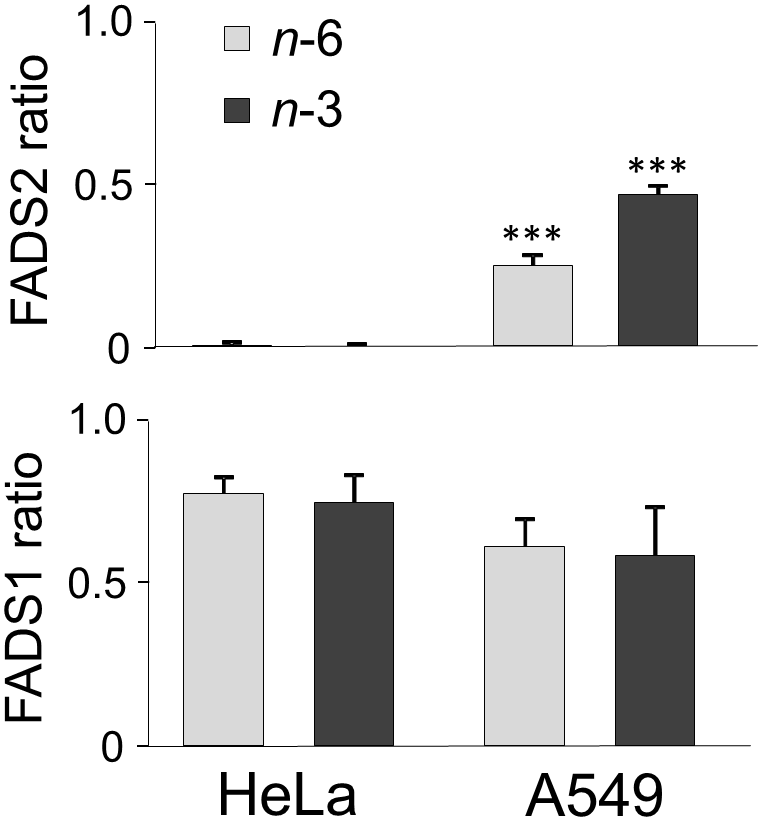
Product-to-substrate ratios as estimates of desaturase activity in HeLa and A549. FADS2 ratio: (C18∶3*n*−6+C20∶3*n*−6)/C18∶2*n*−6 and (C18∶4*n*−3+C20∶4*n*−3)/C18∶3*n*−3 after 24 h (with the second FADS2-dependent step not considered), FADS1 ratio: C20∶4*n*−6/C20∶3*n*−6 and C20∶5*n*−3/C20∶4*n*−3 after 24 h. Increase in FADS2 ratios is significantly different from HeLa (***p≤.001).

Following a 24-h incubation with 33 µM C18∶2*n*−6 or C18∶3*n*−3, the intracellular contents of these fatty acids increased in A549 from 2.0±0.3% and 0.1±0.1% to 14.6±0.7% and 10.2±0.3% of total FAME, respectively (Table 3). Concomitantly, all following intermediates of the *n*−6 and *n*−3 LC-PUFA biosynthesis were detected in abundance up to and including C22-PUFA (*n*−3 PUFA observed after 72 h; Fig. 1B), whose production is essentially dependent on efficient enzyme activity for desaturation (FADS2 and FADS1) and acyl chain elongation (fatty acid elongases ELOVL5 and ELOVL2; Fig. 2) [22]. Ratios reflecting direct FADS2 activity were calculated in A549 at 24 h as 0.24±0.06 for *n*−6 and 0.46±0.1 for *n*−3 (DMSO-ctrl.-corrected p≤.001 *vs.* HeLa, Fig. 3). Taking into account that FADS2 products are themselves substrates for subsequent enzymes of the progressional LC-PUFA biosynthesis, then consideration of the allover product (additional increase in all detected eicosaenoics and docosaenoics in sum) to substrate ratios allows a more comprehensive assessment of the metabolic capacity, particularly as FADS2 catalyzes a second step in the cascade of LC-PUFA synthesis. Thus, *n*−6- and *n*−3-related desaturation ratios increased to 0.65±0.15 for *n*−6 and to 1.17±0.11 for *n*−3 (data not shown) whilst retaining the ratio of *n*−6 to *n*−3 products at approximately 1∶2. These data indicate a substrate preference of FADS2 for *n*−3 over *n*−6 fatty acids in A549 cells, provided that both substrates are initially present in equimolar concentrations, and corroborate previous results obtained from HepG2 (human hepatocellular carcinoma cells) and cardiomyocytes [23], [24].

**Table 3:** Metabolic capacity of HeLa compared to A549.

FADS2required	HeLa		A549		*FA* *treat-ment*	*Cell* *line*	*FA* *treat-ment×cell line*	FADS2passedover	HeLa		A549		*FA* *treat-ment*	*Cell* *line*	*FA* *treat-ment×cell line*
**C18**∶**2** ***n*** **−6** a	**−**	**+**	**−**	**+**	*p = *	*p = *	*p = *	**C18**∶**3** ***n*** **−6** b	**−**	**+**	**−**	**+**	*p = *	*p = *	*p = *
C18∶2*n*−6	2.1±0.3	17.2±1.0	2.0±0.3	14.6±0.7	*<.001*	.*442*	.*014*		2.1±0.3	2.3±0.2	2.0±0.3	1.4±0.2	.*438*	.*402*	.*024*
C18∶3*n*−6	0.0±0.0	0.0±0.0	0.3±0.2	0.8±0.1	.*810*	.*006*	.*002*		0.0±0.0	2.2±0.5	0.3±0.2	5.4±0.8	*<.001*	.*468*	*<.001*
C20∶2*n*−6^c^	0.0±0.1	1.4±0.2	0.1±0.1	1.0±0.1	*<.001*	.*601*	.*008*		0.0±0.1	0.0±0.0	0.1±0.1	0.0±0.1	.*379*	.*554*	.*921*
C20∶3*n*−6	0.3±0.0	0.2±0.2	1.0±0.0	3.5±0.6	.*634*	*<.001*	*<.001*		0.3±0.0	7.8±0.8	1.0±0.0	13.9±1.3	*<.001*	*<.001*	*<.001*
C20∶4*n*−6	1.8±0.3	1.9±0.1	3.9±0.4	8.9±1.3	.*824*	*<.001*	*<.001*		1.8±0.3	8.0±0.8	3.9±0.4	11.7±1.2	*<.001*	*<.001*	.*148*
C22∶4*n*−6	0.1±0.1	0.2±0.0	0.1±0.1	1.8±0.5	.*883*	.*826*	*<.001*		0.1±0.1	1.2±0.1	0.1±0.1	2.1±0.4	*<.001*	.*550*	.*004*
C22∶5*n*−6^d^	0.0±0.0	0.0±0.0	0.0±0.1	0.3±0.1	*1.00*	.*319*	*<.001*		0.0±0.0	0.0±0.0	0.0±0.1	0.3±0.1	*1.00*	.*472*	.*003*
**C18**∶**3** ***n*** **−3** a	**−**	**+**	**−**	**+**				**C18**∶**4** ***n*** **−3** b	**−**	**+**	**−**	**+**			
C18∶3*n*−3	0.1±0.1	16.2±2.1	0.1±0.1	10.2±0.3	*<.001*	.*236*	.*001*		0.1±0.1	0.1±0.1	0.1±0.1	0.0±0.1	.*602*	.*150*	.*813*
C18∶4*n*−3	0.1±0.1	0.0±0.0	0.0±0.1	0.9±0.2	*<.001*	*<.001*	*<.001*		0.1±0.1	2.3±1.0	0.0±0.1	4.7±1.3	*<.001*	.*721*	.*041*
C20∶3*n*−3^c^	0.0±0.0	2.3±0.2	0.0±0.1	1.0±0.1	*<.001*	.*685*	*<.001*		0.0±0.0	0.2±0.2	0.0±0.1	0.0±0.0	.*501*	.*366*	.*308*
C20∶4*n*−3	0.0±0.0	0.0±0.0	0.0±0.0	3.8±0.2	*<.001*	*<.001*	*<.001*		0.0±0.0	9.2±0.2	0.0±0.0	10.1±1.3	*<.001*	.*577*	.*296*
C20∶5*n*−3	0.4±0.1	0.4±0.0	0.5±0.0	6.4±0.4	*<.001*	*<.001*	*<.001*		0.4±0.1	7.3±1.3	0.5±0.0	6.5±1.7	*<.001*	.*006*	.*509*
C22∶5*n*−3	0.8±0.1	0.7±0.2	0.8±0.1	3.1±0.2	*<.001*	*<.001*	*<.001*		0.8±0.1	3.5±0.9	0.8±0.1	2.4±0.8	*<.001*	.*769*	.*199*
C22∶6*n*−3^d^	1.0±0.2	1.0±0.1	1.7±0.1	1.6±0.0^e^	.*808*	*<.001*	.*364*		1.0±0.2	0.9±0.1	1.7±0.1	1.9±0.0^e^	.*036*	*<.001*	.*233*

Fatty acid profiles were determined by GC-FID analysis of cellular lipid extracts and are expressed as % of total FAME. Data represent means ± SD.

a Cells were incubated without (−) or with (+) 33 µM of C18∶2*n*−6 and C18∶3*n*−3, respectively, for 24 h. Both are substrates for FADS2.

b Cells were incubated without (−) or with (+) 33 µM of the FADS2 products C18∶3*n*−6 and C18∶4*n*−3 for 24 h.

c Considered Δ8-desaturation products.

d Synthesis requires upstream a second step of *FADS2*-encoded activity.

e After 72 h, significant increase in A459 to 2.7±0.2 when incubated with C18∶3*n*−3 (FA treatment×cell line: p = .002; see also Figure 1B, peak 14) and to 2.8±0.1 when incubated with C18∶4*n*−3 (FA treatment×cell line: p = .011).

In HeLa, after 24 h incubation with 33 µM of the respective fatty acid, the percentages of C18∶2*n*−6 or C18∶3*n*−3 also increased from 2.1±0.3% and 0.1±0.1% to 17.2±1.0% and 16.2±2.1% of total FAME, respectively (Table 3). However, products directly involving FADS2 activity, such as C18∶3*n*−6 or C18∶4*n*−3, were not detected at the expected concentrations (Fig. 1A), reflected by FADS2 ratios of 0±0 for both *n*−6 and *n*−3 (Fig. 3). In contrast, there was an increase in the respective elongation products C20∶2*n*−6 and C20∶3*n*−3, considered as substrates for the alternative FADS2-catalysed Δ-8 desaturation [11] (Table 3, Figs. 1, 2A). In several cancer cell lines with FADS2 deficiency, such as K562 and MCF7, it has been shown that FADS1 operates in a compensatory manner to produce unusual C20∶3 and C20∶4 of unclear physiological relevance with butylene- instead of the methylene-interrupted double bonds [25], [26]. However, since we detected neither the usual form nor unusual C20∶3 or C20∶4, both C20∶2*n*−6 and C20∶3*n*−3 denoted dead-end products in HeLa. Interestingly, when HeLa were incubated with the Δ-6 desaturation products C18∶3*n*−6 and C18∶4*n*−3 with an aim to passing over this rate limiting step of PUFA biosynthesis, concentrations of all the following metabolites increased within the cellular lipid fraction up to the second step that involves FADS2 activity (Table 3, Fig. 2B). Moreover, FADS1 ratios for both *n*−6 and *n*−3 were similar for A549 and HeLa (Fig. 3).

### *FADS2* expression and sequence analyses

**Figure 4:**
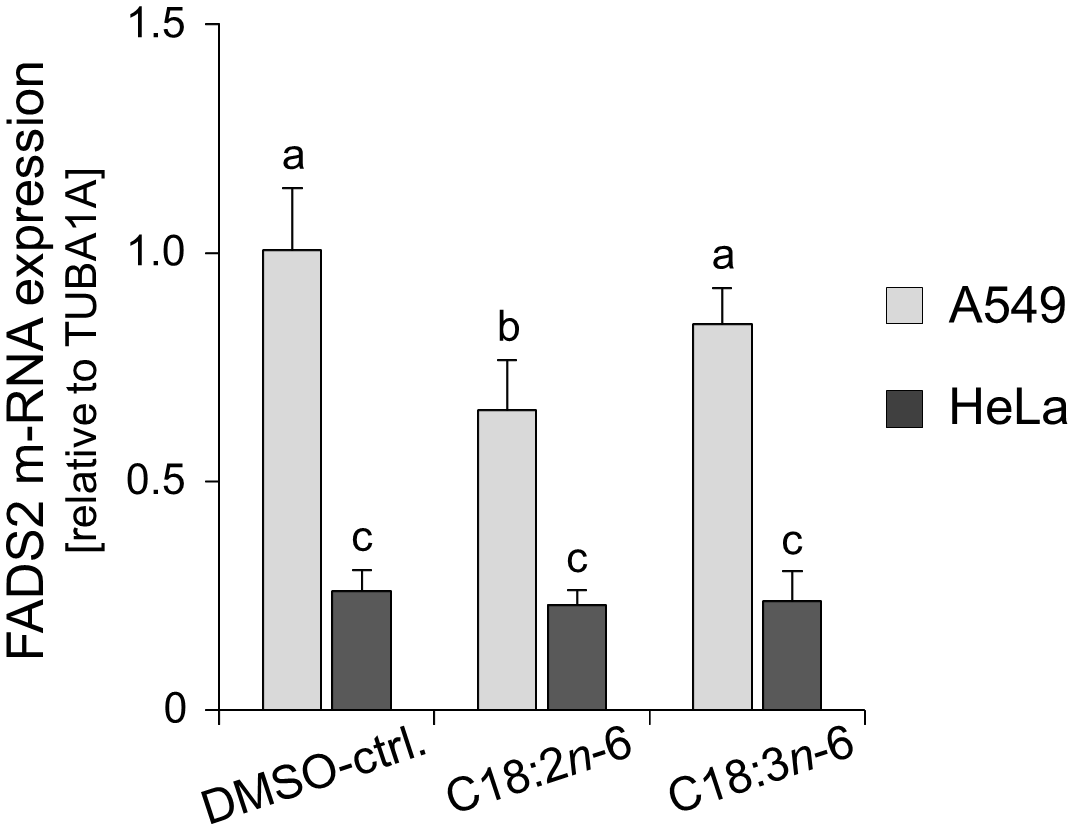
Real-time quantitative expression of *FADS2* mRNA in HeLa and A549. Cells were incubated without (DMSO-treated control) or with 33 µM C18∶2*n*−6 or C18∶3*n*−6 for 24 h. Different letters indicate significant differences: a *vs.* b, and b *vs.* c, p≤.005.

Since firstly, several single nucleotide polymorphisms (SNPs) are associated with decreased FADS2 activity [27], [28] and altered transcriptional regulation [29], and secondly, its gene cluster locus on human chromosome 11q12–13.1 is susceptible to carcinogenic alterations [rev. in 26], we hypothesized that the lacking capacity for Δ-6 desaturation of HeLa resides at the transcriptional level. Therefore, we pursued the following two strategies: first, we compared HeLa with A549 regarding *FADS2* mRNA expression, and second, we proceeded to a *FADS2*-specific sequence analysis of genomic DNA in HeLa. Because previous observations suggest that *FADS2* is regulated by both its substrates and products [30], we performed gene expression analysis in cultures after 24 h incubation with either C18∶2*n*−6 (FADS2 substrate) or C18∶3*n*−6 (FADS2 product). In A549, the amplicon of *FADS2* was detected in relative abundance. Presumably as a negative feedback response, *FADS2* mRNA was down-regulated upon 24 h incubation with C18∶2*n*−6 (p≤.005 *vs.* DMSO-ctrl.) and, to a lesser and non-significant extent, also in response to C18∶3*n*−6 (Fig. 4). In HeLa, the expression level of *FADS2* mRNA was only about a quarter of that in A549 and neither altered by C18∶2*n*−6 nor by C18∶3*n*−6 (Fig. 4). This outcome supports our observation that HeLa cells failed to metabolize C18∶2*n*−6 and C18∶3*n*−3 to their respective Δ-6 desaturation products (Table 3) what indicates lacking FADS2 functionality.

Many transformed cells that are not capable of Δ-6 desaturation have been shown to possess Δ-5 desaturating ability at functional and transcriptional levels [9]. In accordance, the *FADS1* expression levels in HeLa were not different from those detected in A549 (data not shown), what fits with the data obtained from GC-FID analysis (Table 3).

To verify the DNA sequence of *FADS2* in HeLa, all coding exon fragments were cloned, sequenced and compared with the published sequence (GenBank: AP002380). Since we did not find any sequence alteration within the coding region of *FADS2*, we can only speculate about the conditions that led to a loss of FADS2 function in HeLa. Maybe this deficit is the consequence of alterations within the promoter region of *FADS2*, as suggested [31], or post transcriptionally, of unusual protein folding that, as yet, remains elusive.

### Cyclooxygenase-mediated PGE2 formation and expression of CD36

Using the example of PGE2, we further looked at eicosanoid formation, which crucially depends on an efficient upstream biosynthesis of PUFA. We hypothesized that the lacking activity of FADS2 in HeLa consequently resulted in an altered pattern of PGE2 formation from upstream C18∶2*n*−6 as compared with C18∶3*n*−6 as well as compared with A549. In both HeLa [32] and A549 [8], TLR2 stimulation rapidly induces COX-2 through p38 mitogen activated protein kinase (MAPK) and nuclear factor kappa B (NFκB) pathways. Moreover, both HeLa and A549 highly express PGE synthase in response to cytokine stimulation, the enzyme acting downstream from COX-2 to convert the prostaglandin endoperoxide H2 (PGH2), deriving from C20∶4*n*−6, into PGE2 [33]. For induction of cyclooxygenase activity, we mimicked chlamydial infection by TLR2 engagement. Both HeLa and A549 up-regulate TLR2 upon stimulation with microbial components [34]. TLR2 signaling is dependent on the expression of co-receptors. As HeLa lack TLR1 expression and are non-responsive to TLR2/TLR1 receptor pair ligands [6], we used PAM2CSK4 to induce TLR2/TLR6 signaling with subsequent activation of the COX-2 pathway. Stimulation with TNF-α as a TLR-independent COX-2 activator served as positive control.

**Figure 5:**
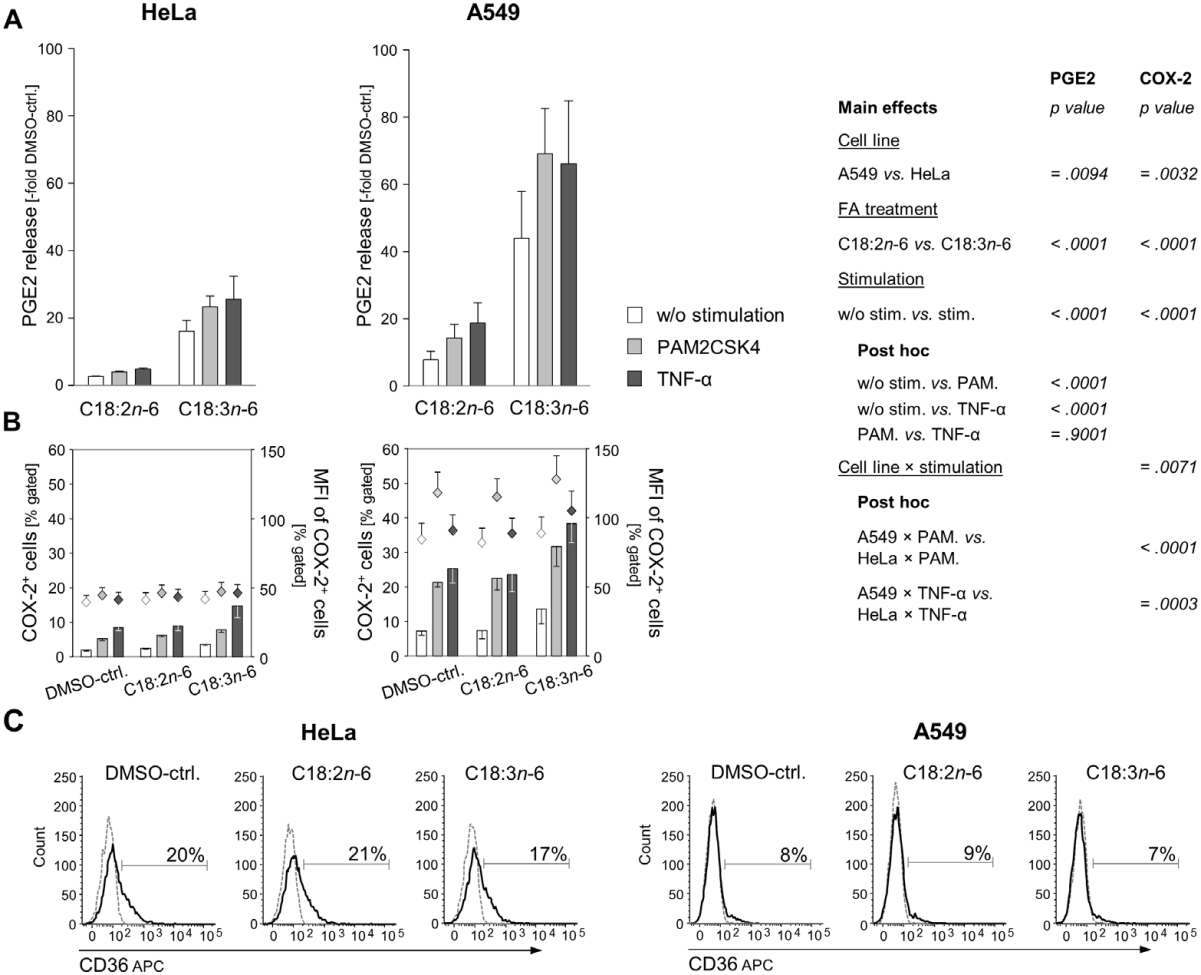
Induction of PGE2 formation and expression of COX-2 and CD36 in HeLa and A549. Cells were incubated without (DMSO-treated control) or with 33 µM C18∶2*n*−6 or C18∶3*n*−6 for 20 h. Subsequently, cells were stimulated with the TLR2 agonist PAM2CSK4 or TNF-α for further 8 h. **A:** PGE2 in supernatants was quantified by EIA technique and is expressed as -fold increase over the unstimulated DMSO-treated control. Means ± SEM. **B:** Percentage of COX-2 positive cells were determined by intracellular staining and flow cytometric analysis. **C:** Representative histograms of HeLa and A549 incubated without (DMSO-ctrl.) or with 33 µM C18∶2*n*−6 or C18∶3*n*−6 for 4 h and stained for CD36. Markers were set in reference to the isotype controls (dotted lines).

In general and in agreement with the literature, PGE2 was well detectable in both cell lines following stimulation procedures. In order to make both cell lines comparable, we related the changes in PGE2 to the respective DMSO-controls. In HeLa, C18∶2*n*−6 elevated the PGE2 release 3-fold compared to the DMSO-ctrl. After preincubation with C18∶2*n*−6, there was only a weak additional increase in PGE2 in response to PAM2CSK4 (4-fold compared to the DMSO-ctrl.) or TNF-α (5-fold compared to the DMSO-ctrl.; Fig. 5A). In contrast, when HeLa were preincubated with C18∶3*n*−6, PGE2 release was enhanced by the factor of 16 and augmented further following either stimulation procedure (23-fold and 26-fold, respectively, over the DMSO-ctrl.; main effect FA treatment: p<.0001). This outcome is in line with our observation that the cascade of LC-PUFA synthesis stops at the level of FADS2 in HeLa (Fig. 2) with consequences for downstream eicosanoid formation. In A549, in both C18∶2*n*−6 and C18∶3*n*−6 pretreatments, PGE2 formation increased in response to PAM2CSK4 and TNF-α, respectively. The increase in PGE2 over the DMSO-control was generally stronger in A549 than in HeLa treatments (main effect cell line: p = .0094; Fig. 5A) and fits with the expression patterns of COX-2 (main effect cell line: p = .0032; Fig. 5B).

After 8 h stimulation, the percentage of A549 cells positive for COX-2 raised approximately 4-fold to 25% (Fig. 5B). The stimulation effect of PAM2CSK4 was comparable with that of TNF-α. In the presence of C18∶3*n*−6, but not C18∶2*n*−6, the percentage of COX-2 positive cells further increased to 30% (PAM2CSK4) and 38% (TNF-α), respectively. The mean fluorescence intensity (MFI) reflecting the COX-2 expression on a per cell basis was highest in response to PAM2CSK4. In contrast to C18∶2*n*−6, C18∶3*n*−6 exerted a stimulatory effect on COX-2 expression itself (main effect FA treatment: p<.0001). We have recently shown that the presence of its substrates sufficed to induce COX-2 [18], what is in line with previous findings [35], [36].

Aiming at elucidating how C18∶3*n*−6 exerts this stimulatory effect itself we measured the expression of the transmembrane scavenger receptor CD36. TLR2/6 signaling is closely linked to CD36 that acts as an accessory co-receptor, sensing and clustering TLR2 ligands, to facilitate TLR2-mediated NFκB activation [37]. But CD36 has also been reported to initiate a downstream cascade upon ligand binding TLR-independently [38]. A main function of CD36, therefore alternatively named fatty acid translocase (FAT), is the transmembrane transport of long-chain fatty acids (LCFA). The degree of unsaturation has previously been suggested to be a determinant for CD36 regulation [39]. Nevertheless, measuring the surface expression of CD36 on both A549 and HeLa revealed no regulatory effect of either C18∶2*n*−6 or C18∶3*n*−6 treatment for 4 h (Fig. 5C) or 24 h (not shown), whereby the expression level of CD36 was generally higher in HeLa than in A549 (Fig. 5C). Hence, of special interest is the finding of Chêne *et al.*, that the number of double bonds of the LCFA the cell is exposed to appears to be critical for the mobilization of C20∶4*n*−6 from membrane phospholipids *via* phospholipase A2 [35].

In HeLa, the basal level of COX-2 was approximately half as much as in A549 (Fig. 5B), what accords to the basal levels of C20∶4*n*−6 (Table 3). Consequently, the percentage of COX-2 positive HeLa cells increased hardly over 10% upon either fatty acid or stimulus treatment (interaction effect cell line×stimulation: p≤.0071; Fig. 5B). It is likely that COX-1 contributed to PGE2 synthesis, because specific inhibition of COX-1 prior to stimulation abrogated the stimulation effect and PGE2 remained on the level detected in the unstimulated fatty acid treatments (not shown). Previous data suggest that inefficiency of COX-2 can partly be compensated by COX-1 activity in HeLa [32]. Nevertheless, COX-1 expression was detected in less than 2% of the cells, was not different between the two cell lines, and remained unaltered upon either fatty acid or stimulus treatment (data not shown).

### Rates of chlamydial infection

**Figure 6:**
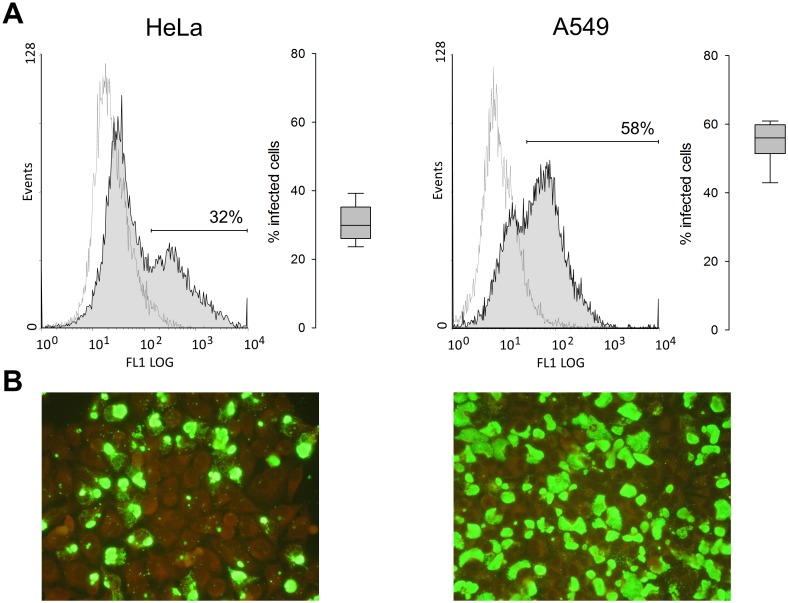
Flow cytometric and fluorescence microscopic assessment of chlamydial infection of HeLa and A549. Cells were inoculated with strain DC15 at MOI of 4 and analysed 48 h p.i. **A:** Representative histograms of fluorescence intensities of infected cells (filled peaks) and uninfected controls (dotted lines) stained with FITC-labeled mAb (BDI168) against *Chlamydia*-specific lipopolysaccharide. Boxplots represent five independent experiments, each done in duplicate. **B:** Cells were stained using the IMAGEN kit. Infection was visualized using fluorescence microscopy at a magnification of ×40.

As a strategy for long-term survival within the host, *Chlamydia* ssp. promote target cell survival in the early phase of infection by inducing upregulation of COX-2 und subsequent PGE2 production. It was shown that blocking of COX-2 reduces the infection rate *in* *vitro* and diminishes the infectious load *in* *vivo*, likely by compromising the completion of the developmental cycle [40]. Fig. 6 shows that infection rates 48 h p.i. with strain DC15 at MOI of 4 were significantly lower in HeLa than in A549 (31±5% *vs.* 55±6% positive cells, as determined by flow cytometry; p = <.001), which is in accordance with weak COX-2 response following stimulation in HeLa (Fig. 5B).

## Conclusion

In conclusion, our data show that HeLa cells differ considerably from A549 at several stages of fatty acid metabolism. The poor metabolic potential of HeLa, mainly concerning FADS2 upstream of COX-2 function, calls into question whether these cells represent a good model to unveil fatty acid or downstream eicosanoid effects in the course of intracellular bacterial infection. Moreover, our findings implicate that it is of basic interest to characterize the metabolic capacity of epithelial cell lines prior to use.
